# Pyrosequencing analysis of bacterial community changes in dental unit waterlines after chlorogenic acid treatment

**DOI:** 10.3389/fcimb.2024.1303099

**Published:** 2024-01-17

**Authors:** Na Li, Qin-Ming Cai, Ni-Ya Hu, Shu-ling Jiang, Fu-Qing Chen, Qiao-Qiao Hu, Fen Yang, Chao-Zhu He

**Affiliations:** ^1^ Department of Stomatology, The First Affiliated Hospital, Jiangxi Medical College, Nanchang University, Nanchang, China; ^2^ Nursing School, Nanchang University, Nanchang, China; ^3^ The First Affiliated Hospital of Nanchang University, School of Public Health, Nanchang University, Nanchang, China; ^4^ The Second School of Clinical Medicine, Southern Medical University, Guangzhou, China

**Keywords:** chlorogenic acid, bacterial community, dental unit waterlines, pyrosequencing analysis, clinical practice-related infections

## Abstract

**Introduction:**

The contamination of dental unit waterlines (DUWLs) poses a significant risk of cross-infection in dentistry. Although chemical disinfectants have been effective in reducing number of bacteria, they do have limitations.

**Methods:**

This study aimed to investigate the potential of chlorogenic acid, a natural substance with broadspectrum antibacterial properties, for treating DUWLs. Over a period of three months, we analyzed the microbial communities in 149 DUWLs samples collected from 5 dental units using high-throughput pyrophosphate sequencing.

**Results:**

The results revealed that chlorogenic acid treatment had a significant impact on the microbial community profile in the DUWLs, with the most significant changes occurring within the first 15 days and stabilization observed in the last 30 days. The predominant genera detected in the samples were Bacteroides, Lactobacillus, Streptococcus, Methylobacterium, and Phreatobacter. Additionally, the relative abundance of certain beneficial bacteria, such as Alloprevotella, Roseburia, and Blautia, increased, while the presence of opportunistic pathogens like Mycobacteria significantly decreased. The functional prediction analysis using the KEGG database indicated a decrease in the pathogenicity of the bacterial community in the DUWLs following chlorogenic acid treatment.

**Discussion:**

This study introduces a novel approach for the prevention and treatment of infections associated with dental care.

## Introduction

According to the US Centers for Disease Control and Prevention (CDC), 64.9% of adults and 84.9% of children in the USA visited dental clinics in 2019 (Liu et al., 2019). However, globally, there were 3.5 billion cases of oral conditions reported in 2017 (Collaborators et al., 2020). A dental unit (DU) is an essential piece of equipment used in oral treatment, which comprises various high-powered tools connected to external pipes and a rinsing system (Porteous et al., 2015). During dental procedures, water flows through these pipes to cool and irrigate dental instruments and surfaces (Siegel and von Fraunhofer, 2002; ODonnell et al., 2005), but studies have shown that they can also become contaminated by various microorganisms due to the high-speed suction and contaminated source water, among other reasons (Spagnolo et al, 2020 ; Szymanska and Sitkowska, 2013; Szymanska et al, 2008; Dallolio et al., 2014; Costa et al., 2016; Alkhulaifi et al., 2020; Castellano Realpe et al., 2020).

The CDC has established infection control guidelines for dental healthcare settings, which recommend a maximum level of 500 CFU/mL of aerobic heterotrophic bacteria in the output water of DUWLs. Yet, studies have consistently shown that the bacterial load in DUWLs output water frequently surpasses this recommended limit, with levels ranging from 10^2^ to 10^8^ CFU/mL (Tuttlebee et al., 2002; Kohn et al., 2004; ODonnell et al, 2006). In some instances, significant amounts of endotoxin have also been detected (Tuttlebee et al., 2002; Kohn et al., 2004; ODonnell et al, 2006; Huntington et al., 2007). The biofilm that accumulates on the tubing of DUWLs periodically sloughs off during procedures, causing the release of planktonic cells and byproducts into the water (ODonnell et al., 2007; Yabune et al, 2008). This phenomenon poses a potential risk of infection. Several cases of infection have been associated with DU output water, including pneumonia and candidiasis (Liu et al., 2019). Controlling the number of colonies in the DUWLs and treating the water is crucial in reducing the risk of cross-infection during dental procedures, particularly as more patients with immunodeficiency are being treated. Additionally, water-pray-related aerosols may also contaminate the dental environment, emphasizing the need for maintaining clean and controlled water systems to reduce the risk of infection (Bentley et al, 1994; Eliades and Koletsi, 2020).

Numerous techniques have been utilized to reduce the colony count and eradicate biofilms in DUWLs (Williams et al., 1993; Mills, 2000). However, many dental practices still face challenges with microbial contamination. To tackle this issue, the Italian Health Ministry developed guidelines aimed at reducing microbial contamination and biofilm formation in DUWLs (Ministry Italian Health, 2015), which have been verified by subsequent studies (Costa et al., 2017; Alkhulaifi et al., 2020; Baudet et al., 2020). Moreover, a study has shown that a combination of chemical disinfectants including 0.02% H_2_O_2_, 0.12% chlorhexidine, 12% ethanol, 1:50 original Listerine, and 2% sodium perborate applied over a four-week period can be effective in achieving the cleaning objectives outlined by the CDC (Pawar et al., 2016). Although chemical disinfectants can be an effective solution, they pose several disadvantages, including corrosion of metal components, pipeline clogging, and the potential for residue build-up that may damage dental restoration materials (Smith et al, 2001; Tuttlebee et al., 2002; Bishara et al., 2005; ODonnell et al, 2006; ODonnell et al., 2007; Percival et al., 2009; Cicciu, 2020). As such, chemical disinfectants may not be the optimal solution for disinfecting DUWLs. Alternatively, chlorogenic acid, a hydroxycinnamic and phenolic acid found in traditional Chinese herbal medicine honeysuckle (Lafay et al., 2006), has broad-spectrum antimicrobial properties, including antifungal and antiviral activity (Zhu et al, 2004; Karunanidhi et al., 2013). Chlorogenic acid has been found to possess bactericidal effects against various bacteria, including *Stenotrophomonas maltophilia* resistant to trimethoprim/sulfamethoxazole (Karunanidhi et al., 2013), *Klebsiella pneumoniae* (Kalinowska et al., 2018), *Helicobacter pylori(* Farzaei et al, 2015 *)*, *Escherichia coli*(Ayseli and Yasemin Ipek, 2016), *Staphylococcus epidermidis*(Fu et al, 2016), and *Staphylococcus aureus*(Sousa et al., 2014). Chlorogenic acid has also been shown to have a variety of biological functions, such as antioxidant, anti-inflammatory, hypoglycemic, antimutagenic, and immune regulatory functions(Miao and Xiang, 2020). Therefore, chlorogenic acid may have potential as a safe and effective method for reducing microbial contamination in DUWLs.

With numerous previous studies conducted on fungi in DUWLs (Zhu et al, 2004; Karunanidhi et al., 2013), we here conducted a study to examine the effectiveness of chlorogenic acid treatment in eliminating microorganisms in DUWLs. A total of 149 samples were collected from DUWLs and subjected to bacterial culture and pyrosequencing of the V3/V4 regions in bacterial 16S rDNA from DNA samples. By comparing the community diversity and richness of microorganisms before and after the chlorogenic acid treatment process, we were able to provide valuable new insights into the application of chlorogenic acid as a safe and effective treatment for eliminating microorganisms in DUWLs. To our knowledge, this study is the first to investigate the use of chlorogenic acid in this manner.

## Experimental procedures

### Dental units

The 5 DU used for more than 2 years were chosen from the Department of Stomatology of the First Affiliated Hospital of Nanchang University. The mains source water for these DU was the municipal water and all of them without disinfection before this study.

### Disinfectant

The high-speed handpiece water of 5 DU was used as the intervention object. The disinfection intervention was carried out after the end of the treatment on working days (Monday to Saturday). Chlorogenic acid is purchased from Xian Foreview Bio-tech Co.,Ltd, with product batch number 20210115. To prepare a chlorogenic acid solution with a concentration of 0.025g/mL, add 1.25 grams of chlorogenic acid to 50ml of distilled water. Maintain the test pipeline in a water-free state before measurement. Fill a 50 mL distilled water into the water storage bottle, switch to “water supply from the water storage bottle.” After the water flows out from the test pipeline, remove the water storage bottle and measure the remaining water in the bottle to be 30 mL. Determine that 20 mL of water is required from the water storage bottle to the outlet of the test pipeline. To ensure that the disinfectant fills the test pipeline during the intervention process, the amount of disinfectant in this study is increased to 50 mL.

To ensure the test pipeline remains dry before disinfection, inject 50 mL of chlorogenic acid solution into the water storage bottle and switch to “water supply from the water storage bottle.” After draining the chlorogenic acid liquid from the water storage bottle, remove the bottle, turn off the power, and let it sit overnight. Periodically monitor the disinfection effect of the water storage bottle. The next day, before starting work, drain the chlorogenic acid liquid from the pipeline and switch to water supply from the water source. Rinse the pipeline for 3 minutes.

### Sample collection

In order to prevent microbial contamination of the samples, before taking the samples, the staff carried out hand hygiene steps, used aseptic gloves during the whole sampling process, disinfected the high-speed outlet twice with 75% alcohol, after drying, water was flushed for 2 minutes and used a sterile bottle for sampling. Here, 510 mL samples were collected each time, divided into 2 parts immediately after sampling. Between them, 10 mL water sample was for bacteria culture. The colony counts are compared with the CDC, recommend a maximum level of 500 CFU/mL. And another 500 mL sample was filtered through a 0.22 μm membrane, which was frozen and stored in a refrigerator at -80℃ for centralized inspection. In order to ensure that the quality of the sample met the standards, we used the same staff during the sample collection process, and the staff received prior training in the associated methods and operations. The last sampling day was the third month of chlorogenic acid intervention. The water in the DU was sampled daily during the first week and every Tuesday and Friday thereafter, before the start of the morning diagnosis and treatment activities. On the Friday of the 7th week, there was a malfunction with dental chair 1 which led to an unsuccessful sampling attempt. As a result, there was one less sample in the second group for that week. Likewise, on the Friday of the 11th week, all five dental chairs failed to successfully collect samples since the department was closed. A total of 149 samples were collected, and samples from the same week were grouped together. These groups were further divided into 14 groups, representing a period of 14 weeks.

### Bacterial culture

The 10 mL water sample was thoroughly shaken and mixed, and 1mL was aspirated and poured into 9 cm diameter blood AGAR plate. The blood AGAR plate was evenly coated with an L-shaped glass rod, and then incubated in a 37°C incubator for 48 h. The number of colonies on the plate was counted and the total number of bacteria in the water sample was calculated. Water and biofilm samples were processed for microbiological culture within 30 min of collection. Serial 10-fold dilutions (up to 10−5) of the samples were prepared in sterile PBS and plated on various selective and non-selective culture media (Hussain et al., 2022). The colony number obtained from some samples after bacterial culture was found to be excessively high. Therefore, we have introduced two criteria to address this issue: specific numerical values and non-specific numerical values.

### DNA extraction

After grinding the filter membrane with a grinding stick, the genomic DNA of the sample was extracted using the MagPure Soil DNA LQ Kit (Magan) according to the instructions provided with the kit. The concentration of the DNA was verified using a Nano Drop system and agarose gel and finally uniformly quantified to 50ng/μL. The genomic DNA was used as template for PCR amplification (94 °C for 5 min, followed by 26 cycles at 94 °C for 30 s, 56 °C for 30 s, 72 °C for 20 s and a final extension at 72 °C for 5 min) with the barcoded primers and Tks Gflex DNA Polymerase (Takara). The 30μL reaction mixture contained 1 μL of 5pmol/μL 343 F and 798 R primers, 15μL 2× Gflex PCR Buffer, 0.6μL Tks Gflex DNA Polymerase (1.25 U/μL) and 1μL template DNA, and the remaining volume was made up with water. For the bacterial diversity analysis, the V3-V4 variable regions of 16S rRNA genes were amplified with the universal primers 343 F (5- TACGGRAGGCAGCAG -3) and 798 R (5- AGGGTATCTAATCCT-3) (Nossa et al., 2010).

### PCR amplification and library construction

The amplicon quality was assessed through visualization using gel electrophoresis, purified with AMPure XP beads (Agencourt) and amplified for another round of PCR (94℃ for 5 min, followed by 7 cycles at 94℃ for 30 s, 56℃ for 30 s, 72℃ for 20 s and a final extension at 72℃ for 5 min). The 30μL reaction mixture contained 15μL 2× Gflex PCR buffer, 0.6μL or 1μL of Adapter I5 and Adapter I7, respectively, and 1μL of the first PCR product, and the remaining volume in the system was made up with water. After being purified with the AMPure XP beads again, the final amplicon was quantified using a Qubit dsDNA assay kit. Equal amounts of purified amplicon were pooled for subsequent sequencing.

### Bioinformatic analysis

Raw sequencing data were in FASTQ format. Paired-end reads were then preprocessed using Trimmomatic software(Bolger et al, 2014) to detect and remove ambiguous bases(N). We also trimmed low-quality sequences with average quality score below 20 using a sliding window trimming approach. After trimming, paired-end reads were assembled using FLASH software (Reyon et al., 2012). The parameters used in assembly are as follows: 10bp of minimal overlapping, 200bp of maximum overlapping and 20% maximum mismatch rate. Regarding the sequences, reads with ambiguous, homologous sequences or below 200bp were abandoned. Reads with 75% of bases above Q20 were retained. Then, chimeric reads were detected and removed. These two steps were achieved using the QIIME software (version 1.8.0) (Caporaso et al., 2010). Clean reads were subjected to primer sequence removal and clustering to generate operational taxonomic units (OTUs) using Vsearch software with a 97% similarity cutoff (Rognes et al., 2016). The representative read of each OTU was selected using the QIIME package. All representative reads were annotated and subjected to BLAST searching of the Silva database version 123 (16S rDNA) using an RDP classifier (confidence threshold of 70%) (Wang et al., 2007).

Based on summarized results, bioinformatic analyses were carried out. QIIME software was used for alpha and beta diversity analysis. The microbial diversity in samples was estimated using the alpha diversity that include Chao1 index, observed species, PD whole tree and Shannon index. The Bray Curtis matrix performed by R package was used for Bray Curtis to estimate the beta diversity. In addition, we conducted random forest analysis to identify the key components that can determine the differences between groups.

PICRUSt functional prediction was based on 16S sequencing data annotated in the Greengenes database (DeSantis et al., 2006). PICRUSt software was used to predict the composition of known microbial gene functions, so as to calculate the functional differences between different samples and groups (Langille et al., 2013).

### Statistical analysis

Byssal thread counts were analyzed by SPSS software (SPSS 22.0), and one-way ANOVA and LSD test were used to evaluate the significant difference. R package was used to analyze the significant differences between different groups using Kruskal-Wallis statistical test. The linear discriminant analysis effect size (LEfSe) method was used to compare the taxonomy abundance spectrum. Significant difference was considered as *p*< 0.05.

## Results

### Bacterial contamination status before and after intervention

Before conducting this experiment, the research group conducted an evaluation of the intervention effect of chlorogenic acid, 3%H_2_0_2_, and distilled water on DUWLs for a period of 89 days. The evaluation of the intervention effect was conducted daily before the intervention, in the first week after the intervention, and every Tuesday and Friday thereafter. The results showed that there was no statistical difference in the intervention effect between the chlorogenic acid group and the 3%H_2_0_2_ group, but both groups had a statistical difference compared to the distilled water group (**Supplementary Table 2**). Before intervention, the bacterium of 5 dental units high-speed handpiece water exceeded the standard, until 58 days after the intervention, the number of colonies in the dental chair was<500 CFU/mL (**Supplementary Table 1**).

### Sequencing data of DUWLS samples

The relative bacterial colonization of the 5 DU was monitored using quantitative PCR based on the 16S rRNA gene. A total of 149 samples were included in the project, and after quality control, the data volumes of clean tags ranged from 28309 to 73586 with an average of 66320. We removed chimeras in the clean tags and finally obtained valid tags for subsequent OTU partitioning. The data volumes of valid tags ranged from 22572 to 67857, with an average of 59746. The average lengths of the valid tags ranged from 312.01 to 415.53bp and the OTU numbers of each sample ranged from 1651 to 3678.

**Figure 1:**
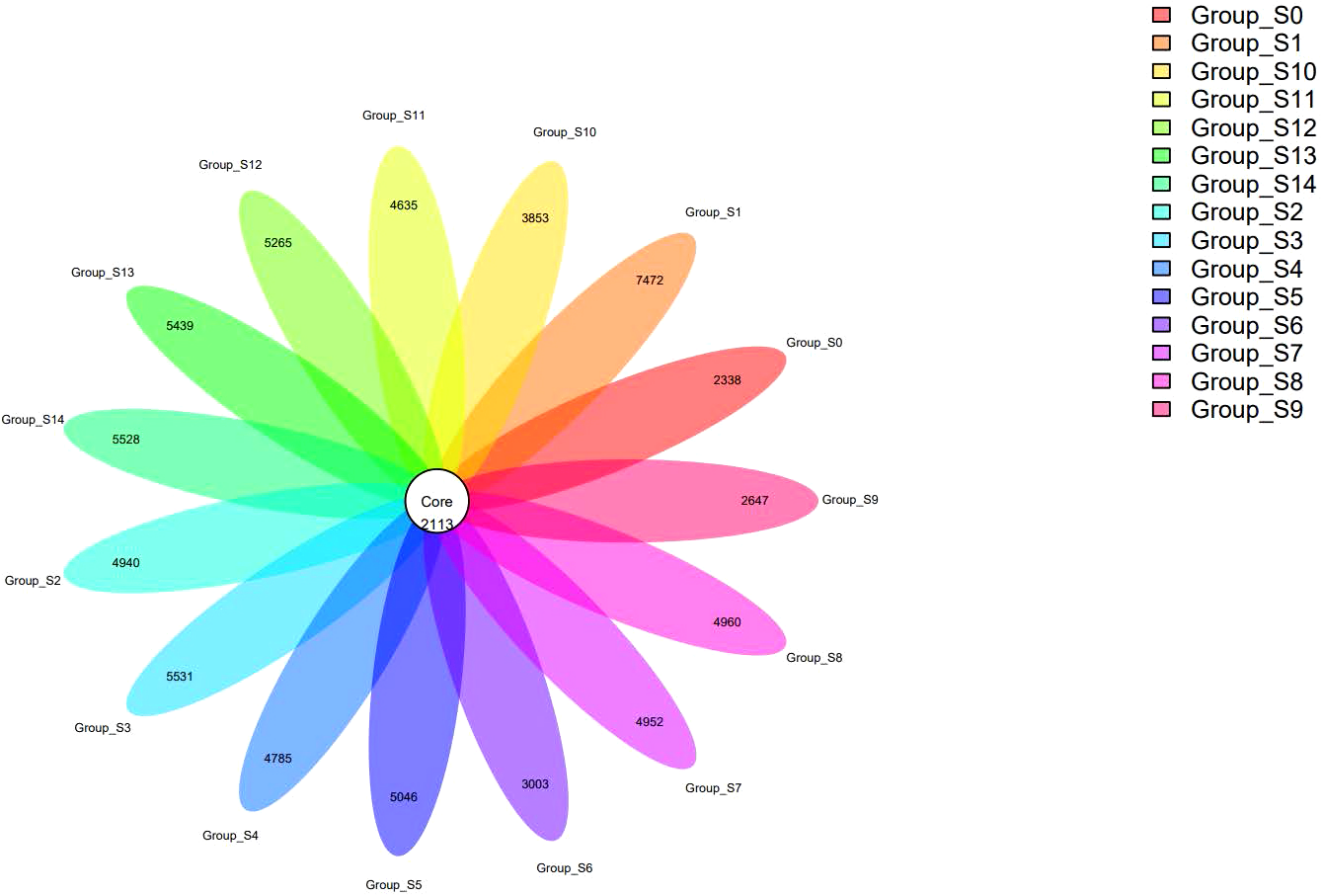
A flower plot representing the shared and unique OTUs among groups.

We conducted the analysis in groups with the numbers of OTUs in all groups ranging from 4451 to 9585. A flower plot analysis was conducted to identify the shared OTUs (**Figure 1**). Among all OTUs, 2113 were shared by all groups, indicating the presence of a continuous core bacterial community present in the DUWLs during the chlorogenic acid treatment process. However, there were a considerable number of different OTUs among each group, indicating that some bacterial communities were changing under the intervention with chlorogenic acid while the continuous core bacterial community persisted. We used the statistical tests to examine the differences in the project samples. The ANOVA algorithm results revealed 1818 different OTUs, 243 different genera and 10 different phyla. The Kruskal-Wallis algorithm results revealed 1877 different OTUs, 316 different genera and 11 different phyla.

### Changes in community richness and diversity indices of DUWLs under the intervention with chlorogenic acid

To ensure a fair comparison of sample diversity, the unified sampling depth approach was employed to mitigate any variations caused by this factor. The resulting diversity indices for each sample were compiled into a table for analysis. By using PD whole-tress, Shannon, chao1, and observed species indices at a 95% sequence similarity cutoff (**Supplementary Figures 1A-D**), the bacterial community diversity and richness in each DUWLs were assessed. The results from all four indices indicate that the group treated with chlorogenic acid exhibited a higher richness and variability when compared to the control group, with statistical significance observed for PD whole-tree, chao1, and observed species indices (*P* < 0.01), as well as the Shannon index (*P* < 0.05). Simpson indices were also calculated, and the results also showed relatively more richness and higher variability, although not statistically significant (*P* > 0.05). In addition, Specaccum species accumulation curve and rank abundance analyses were conducted, and the results showed that the sampling was adequate. The regression curve of the β diversity distance was based on the Bray-Curtis distance between two pairs of samples calculated using the vegan package. The distance from the end point was selected, and ggplot2 was used to visualize the scatter plot indicating the relationship between the distance and time variables. Time nodes were grouped according to 15-day intervals, and a fitting curve was added to allow observation of the change rule. Altogehter, the above results suggest that the chlorogenic acid intervention is effective in increasing the richness of the microbial community in the DUWLs but decreasing its diversity.

### Community structure distribution under chlorogenic acid intervention

**Figure 2:**
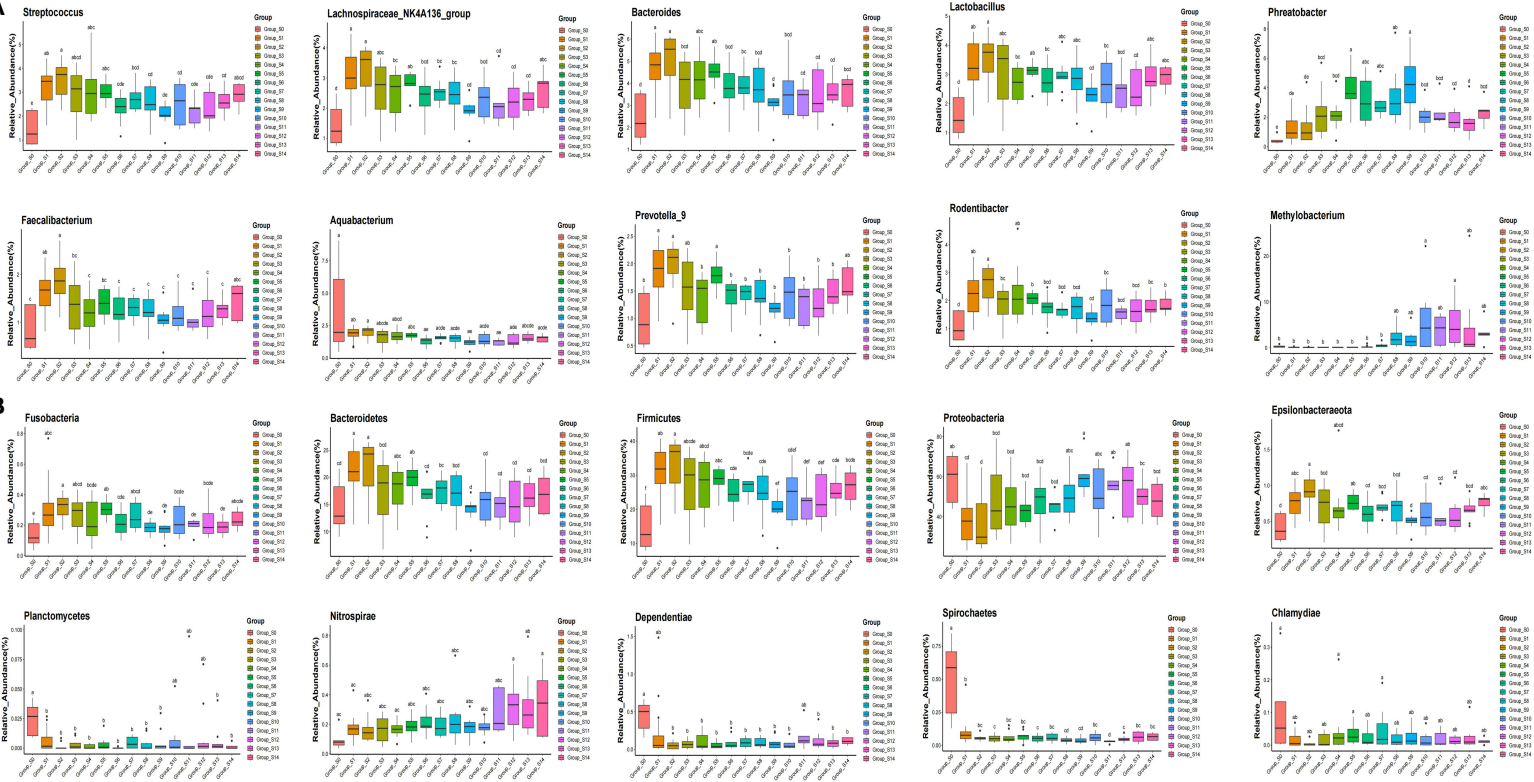
Boxplot of top ten phyla and genera showing statistically differences in response to chlorogenic acid treatment. Different colors represent different groups of samples, and the ordinate represents log conversion values of the relative abundance of species. Species with zero relative abundance in a group are not displayed. **(A)** groups represent top ten genera with statistically differences; **(B)** groups represent top ten phyla with statistically differences. Using the significant difference letter labeling, significant differences between groups are identified.

**Figure 3:**
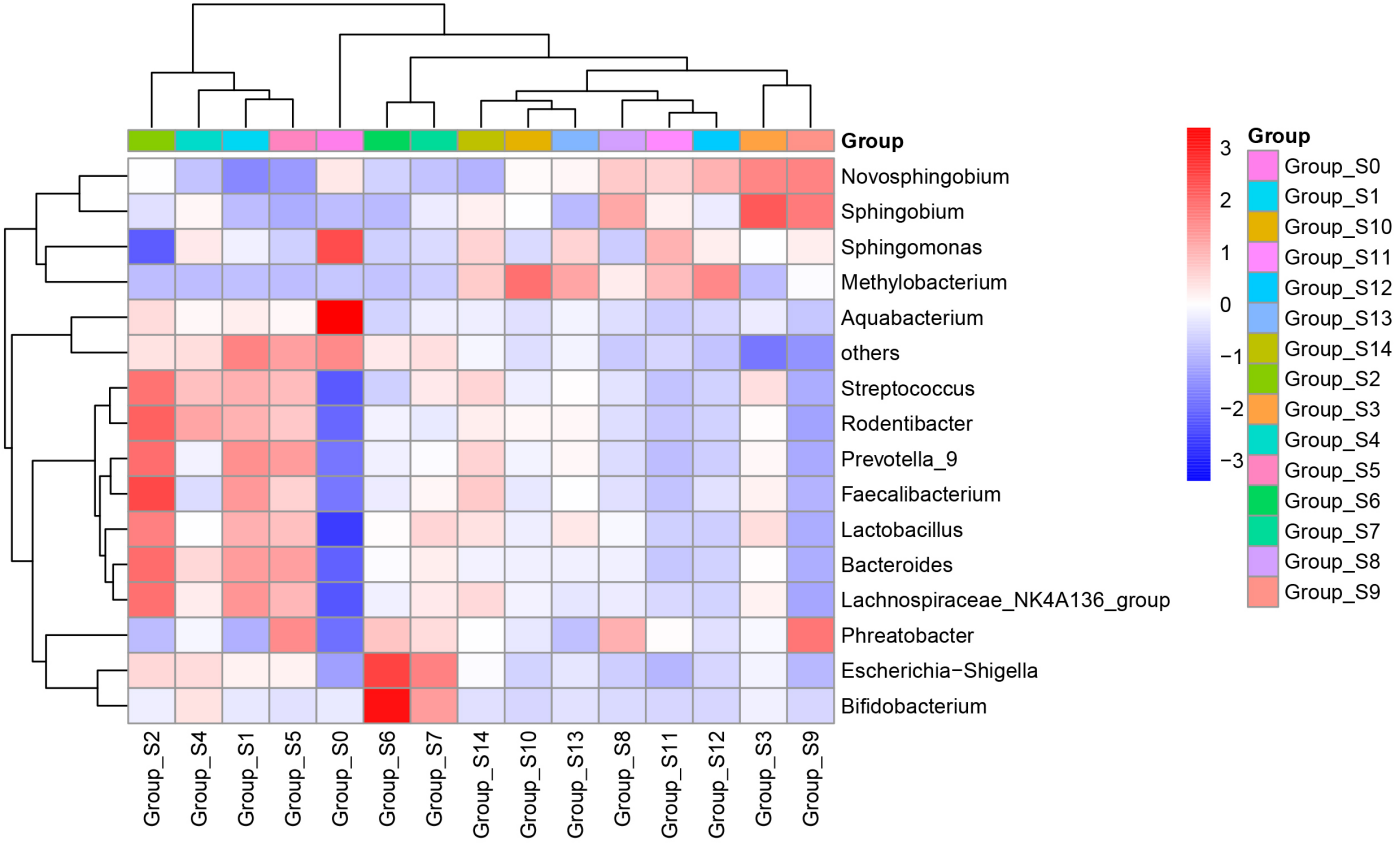
Heatmap of relative abundances of genera. The abscissa represents groups based on sampling times, and the ordinate represents relative abundances of genera. Blue indicates low relative abundance and red indicates high relative abundance.

The pyrophosphate sequencing data were analyzed based on taxonomical classification to identify alterations in bacterial community composition following chlorogenic acid treatment. At the phylum level, we identified 24 phyla, which collectively accounted for more than 99.9% of the relative abundance in each sample. Among these, *Proteobacteria* (49.57 ± 6.1%), *Firmicutes* (24.94 ± 4.2%), *Bacteroidetes* (16.91 ± 2.2%), and *Actinobacteria* (5.65 ± 1.2%) were the dominant phyla, contributing to over 96% of the relative abundance in each sample (**Supplementary Figure 2**). Notably, we observed that following chlorogenic acid treatment, the relative abundances of ten phyla significantly decreased (e.g. *Proteobacteria*, *Spirochaetes*, *Chlamydiae*), while the relative abundances of five phyla increased (e.g. *Fusobacteria*, *Bacteroidetes, Firmicutes, Epsolonbacteraeota* and *Nitrospirae*) (**Figure 2**). At the genus level, we found *Bacteroides*, *Lactobacillus*, *Streptococcus*, *Lachnospiraceae_NK4A136_group*, *Phreatobacter*, *Rodentibacter*, *Methylobacterium*, *Aquabacterium*, *Prevotella_9*, and *Faecalibacterium* to be the top ten genera with significant differences due to chlorogenic acid intervention (*P* < 0.01, *FDR_P* < 0.01). Compared to the control group, the relative abundances of these genera were higher in the chlorogenic acid treatment group. However, the relative abundance of *Methylobacterium* showed an initial decline followed by an increase. We depicted the relative abundances of genera among groups using a heatmap (**Figure 3**). Furthermore, we found several probiotic genera (e.g.*Ruminococus_2*, *Alloprevotella*, *Roseburia*, *Blautia*) that significantly increased following chlorogenic acid treatment. Conversely, *Mycobacterium* and *Aquabacterium* displayed significant reductions in relative abundance (Kruskal–Wallis difference statistics: *P* < 0.01, *FDR_P* < 0.05), with many species of *Mycobacterium* known to be of pathogenic relevance to human health (Tortoli, 2014).

### Correlation and model prediction analysis of DUWLS bacterial community

**Figure 4:**
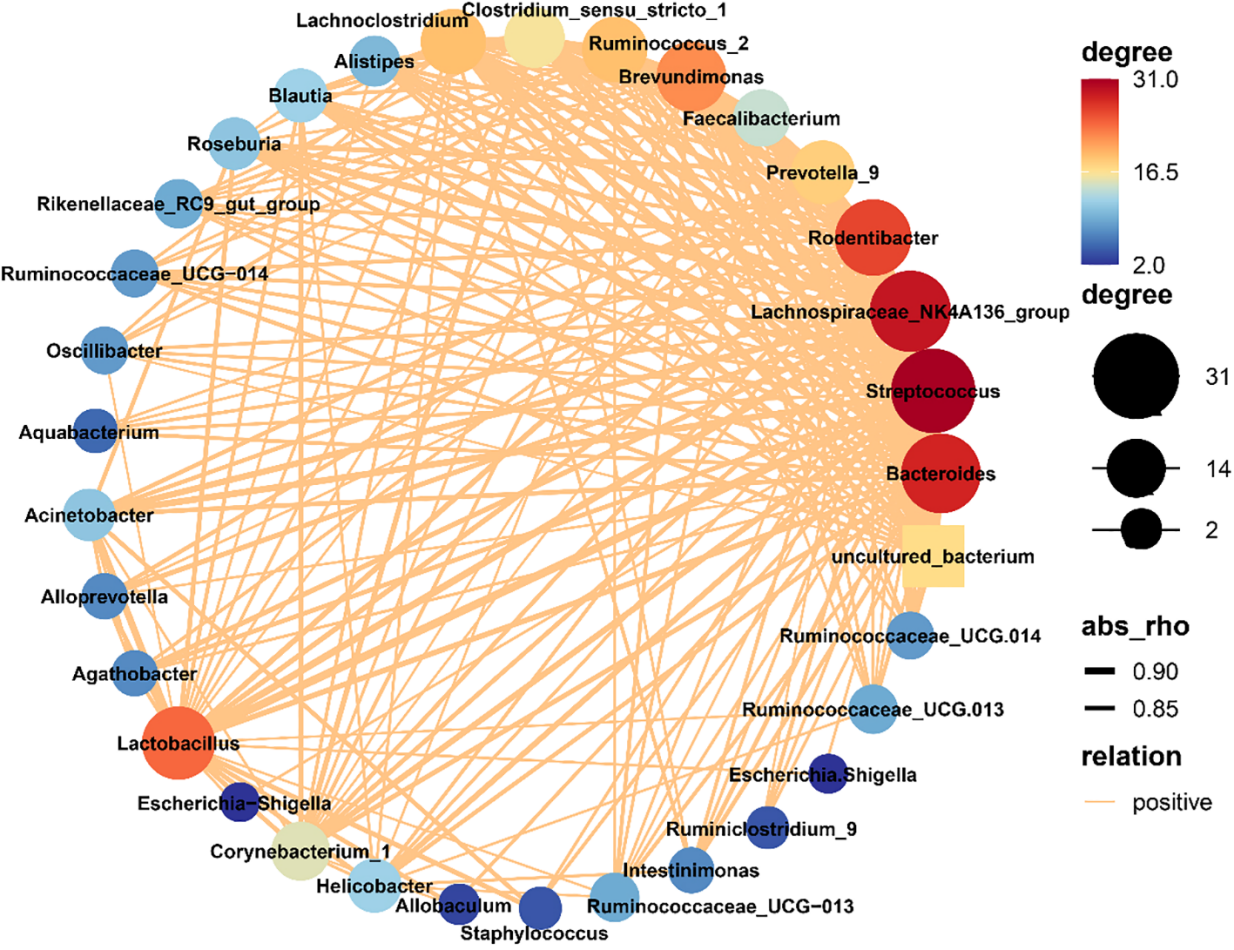
Network of bacterial communities. The size of a node in the figure is indicative of the species abundance, and different colors indicate different species. The colors of the lines indicate positive (red) and negative (green) correlations. The thickness of the line is indicative of the degree of Pearson correlation; i.e., the thicker the line, the higher the correlation between species, and more lines for a given species, the more other species with which it is closely related.

In order to assess the mutual relationship between species within or between sample groups, the Spearman correlation coefficient is calculated based on the relative abundance of species samples. Furthermore, a species interaction network is constructed using visualization software to display the interrelationship between species. This network graph showcases the correlation of species at different taxonomic levels under the influence of chlorogenic acid treatment. We selected the top 50 species in terms of total abundance for further analysis and explored the relationships between them using Spearman correlation coefficients. The resulting species interaction network was constructed using visualization software, highlighting the species meeting the criteria of Spearman Coefficient >0.8 and *P*<0.01 (**Figure 4**). The network diagram was mainly composed of *Firmicutes*, *Bacteroides* and *Proteobacteria*. Notably, *Staphylococcus*, *Bacteroides*, *Lachnospiraceae_NK4A136_group*, *Lactobacillus* and *Brevundimo*nas were identified as the most interconnected genera. Furthermore, we used the 30 genera with the highest relative abundances and the R-packet as a basis for random forest analysis to determine the importance of each species (**Supplementary Figure 3**). The top four genera that determined differences were *Methylobacterium*, *Bdellovibrio*, *Phreatobacter* and *Undibacterium*, as determined by random forest analysis.

### Picrust analysis of DUWLS bacterial community

**Figure 5:**
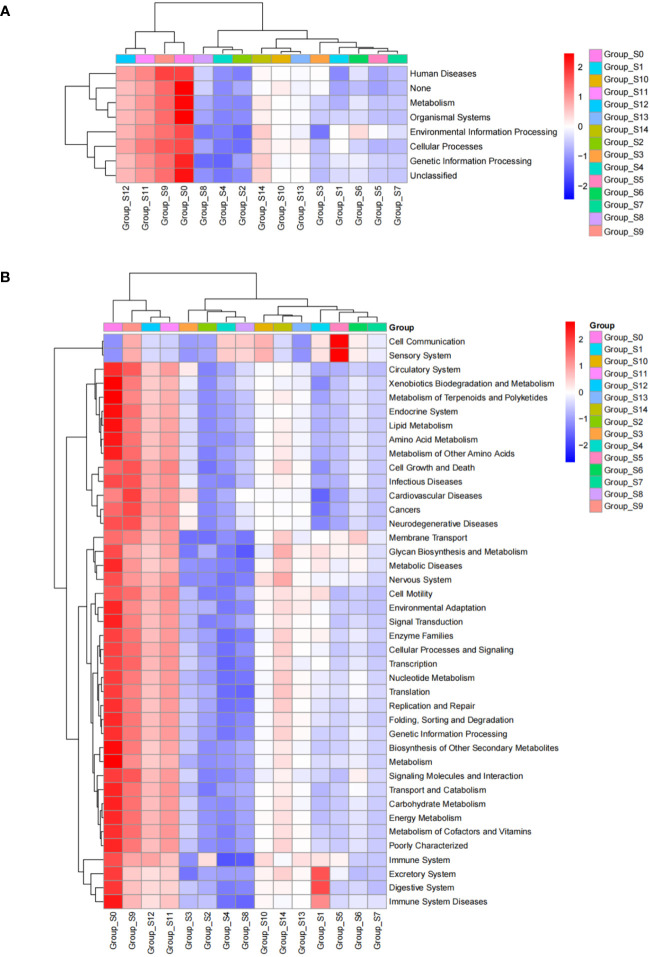
KEGG_L1 **(A)** and KEGG_L2 **(B)** difference results cluster heatmap. The abscissa represents groups based on sampling times, and the ordinate represents KEGG groups. Blue indicates low relative abundance of COG and red indicates high relative abundance of COG.

**Figure 6:**
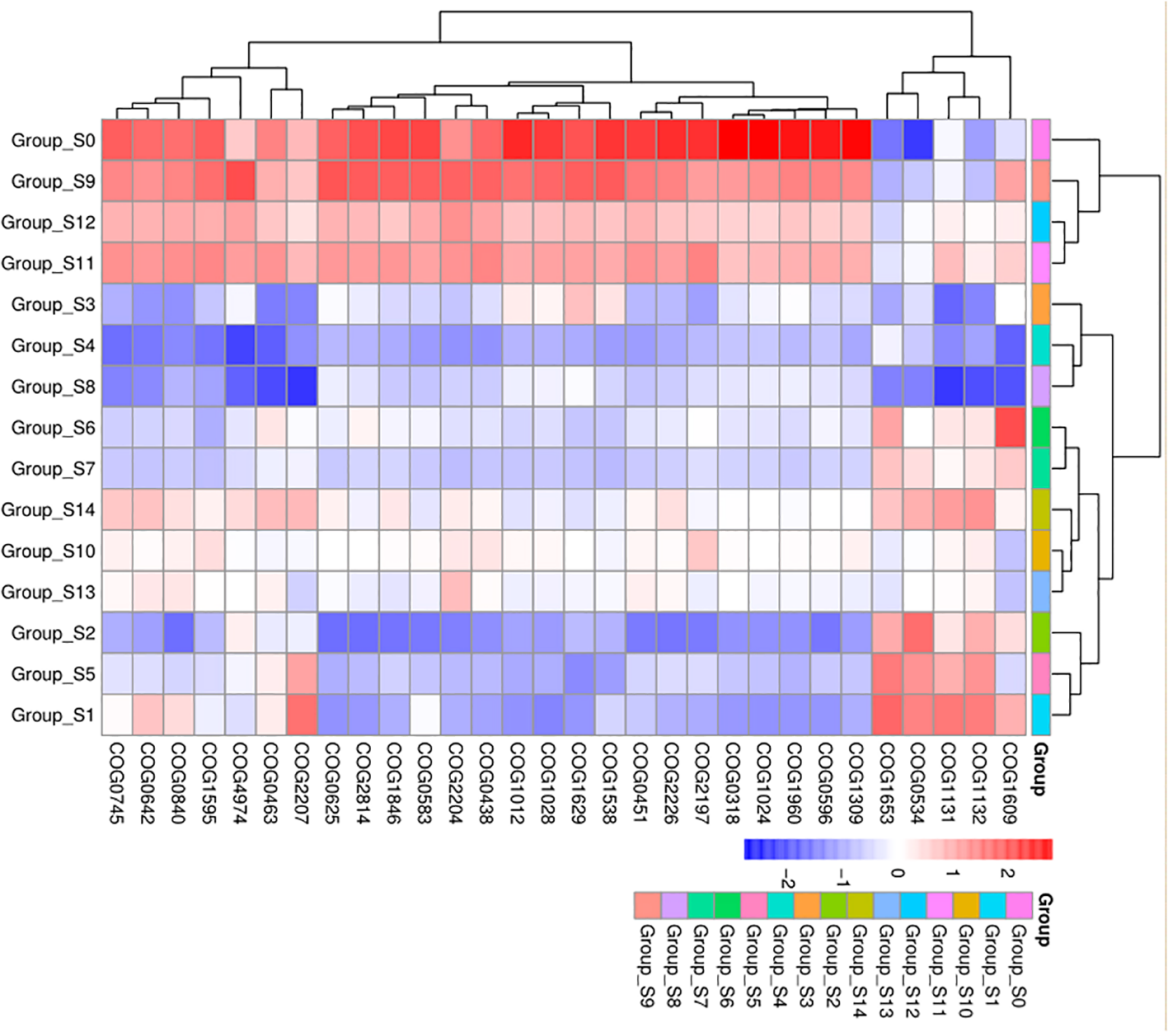
COG difference results cluster heatmap. The abscissa represents groups, and the ordinate represents COG groups. Blue indicates low relative abundance of COG, and red indicates high relative abundance of COG.

We analyzed predicted Kyoto Encyclopedia of Genes and Genomes (KEGG) results on three levels and generated a heatmap diagram of the differences observed between groups, shown in **Figure 5** and **Supplementary Figure 4**. We detected a total of 8 functional differences at the KEGG_L1 level, 37 at the L2 level, and 235 at the L3 level. At the KEGG_L1 level, the control group was clustered into human disease functions compared to the chlorogenic acid treatment group. At the KEGG_L2 level, the control group was significantly clustered into immune system disease functions. Furthermore, using statistical differences indicated by the Kruskal-Wallis algorithm, we calculated the predicted COG results and formed a heatmap diagram using the top 30 results showing the highest significant differences (**Figure 5**). Our results revealed that the relative abundances of COG1309, COG0596, COG1960, COG1024, COG0318, COG2197, and COG2226 were higher in the control group, while that of COG0534 is significantly lower in the control group (**Figure 6**).

## Discussion

In this study, we investigated the potential use of chlorogenic acid, a natural substance renowned for its broad-spectrum antibacterial properties, in the treatment of DUWLs. Our analysis involved the evaluation of 149 DUWLs samples to assess number of bacteria and microbial community before and after the intervention. Bacterial culture and high-throughput pyrophosphate sequencing were employed for this purpose. The study yielded three significant findings. Firstly, prior to the intervention, the number of number of bacteria in the high-speed handpieces of the five dental chairs exceeded the standard limit significantly. Subsequent to the intervention with chlorogenic acid, a decrease in the number of number of bacteria was observed, although some fluctuations were noted. It is recommended to continue the intervention over a specific duration to maintain water quality. Secondly, the study revealed a high bacterial richness and diversity in DUWLs, with *Proteobacteria*, *Firmicutes*, *Bacteroidete*s, and *Actinobacteria* identified as the dominant phyla. This finding is consistent with previous studies demonstrating that *Proteobacteria* and *Actinobacteria* are the predominant phyla in DUWLs (Costa et al., 2015). Additionally, we also detected *Firmicutes* and *Bacteroidetes*, albeit with lower percentages. The main genera detected were *Bacteroides*, *Lactobacillus*, *Streptococcus*, *Methylobacterium*, and *Phreatobacter*. Following the intervention with chlorogenic acid, a decrease in the relative abundance of pathogenic microorganisms, such as *Methylobacterium* and *Mycobacterium*, was observed. Lastly, the intervention with chlorogenic acid demonstrated a reduction in the number of bacteria within DUWLs. These research findings can provide guidance for the clinical management of microorganisms in DUWLs and establish the groundwork for potential use of chlorogenic acid as a disinfectant for DUWLs. The bacterial amount of chlorogenic acid treatment group was less than control group significantly. In addition, the composition of the chlorogenic acid treatment was different from control group. In general, the relative composition of *Bacteroides, Lactobacillus*, *Streptococcus*, *Lachnospiracea*, *Phreatobacter*, *Rodentibacter*, *Methylobacterium*, *Aquabacterium*, *Prevotella*, and *Faecalibacterium* was higher in chlorogenic acid treatment group. Further research and application of chlorogenic acid as a disinfectant in DUWLs maintenance hold promising prospects for enhancing water quality and infection control in dental healthcare settings.

The results of the study illustrate that there were fluctuations observed in the number of number of bacteria within DUWLs immediately after the intervention with chlorogenic acid. It took a duration of 58 days for the colony count within the samples to consistently meet the standards recommended by the CDC, consistently remaining below the threshold of 500 CFU/mL. Further analysis suggests that this instability may be attributed to the continuous shedding of biofilm within DUWLs during the initial stages of the intervention.

The analysis of the microbial community of DUWLs during the intervention revealed the presence of core number of bacteria, accompanied by a decrease in microbiota diversity but an increase in abundance. This indicates that the relative abundance of most bacteria in the dental units waterlines decreases after treatment with chlorogenic acid, such as *Mycobacteria*, while the relative abundance of specific types of bacteria, such as *Lactobacillus* and *Methylobacterium*, increases.

Through random forest analysis, the key species that were found to determine the differences in the bacterial community were identified as *Methylobacterium* and *Bdellovibrio*. Furthermore, the distribution of the community structure highlighted *Methylobacterium* as one of the top 10 bacteria with the most significant variance in abundance following treatment with chlorogenic acid.

*Methylobacterium*, known as a common opportunistic pathogen within building water supply systems (Langille et al., 2013), has been reported to exhibit resistance to various disinfectants, including 1% benzalkonium chloride and chlorine-based disinfectants (Furuhata and Koike, 1993; Yano et al., 2013). This bacterium is capable of thriving and surviving in tap water for extended periods of time. Its propensity for colonization and biofilm formation is attributed to its hydrophobic characteristics (Szwetkowski and Falkinham, 2020).

Studies have documented that *Methylobacterium* can cause respiratory infections, bloodstream infections, and catheter-related bloodstream infections (CRBSI) (Langille et al., 2013). Consequently, the presence and differential abundance of *Methylobacterium* within DUWLs following chlorogenic acid treatment indicate the importance of targeting and controlling this opportunistic pathogen to minimize the risk of associated infections.

In this study, we observed a decreasing trend in the relative abundance of *Methylobacterium* during the first 6 weeks of intervention. However, starting from the 7th week, it began to increase and reached a peak in the 10th week, before decreasing again, although still higher than the levels observed in the first 6 weeks (**Supplementary Figure 5**). Previous research has highlighted the importance of dry storage after disinfection of flexible endoscopes in preventing recontamination with waterborne microorganisms, including *Methylobacterium*(Szwetkowski and Falkinham, 2020). We speculate that chlorogenic acid may have inhibitory effects on the growth of *Methylobacterium*, both in planktonic bacteria and biofilms. However, due to the challenge of maintaining complete dryness in DUWLs and the current contamination of hospital tap water by *Methylobacterium*(Szwetkowski and Falkinham, 2020), fluctuations in the abundance of *Methylobacterium* in DUWLs may occur. It is important to note that this study did not assess the source water, limiting our ability to accurately interpret these results in relation to *Methylobacterium* dynamics.

*Bdellovibrio* is a Gram-negative bacterium that serves as a predator of other bacteria. It controls pathogenic bacteria by parasitizing and lysing the host (Sockett, 2009; Kuru et al., 2017; Duncan et al., 2019). In this study, we observed an increase in the abundance of *Bdellovibrio* after intervention with chlorogenic acid (**Supplementary Figure 6**). Several studies have demonstrated that *Bdellovibrio* can inhibit Gram-negative bacteria in the oral cavity, thereby preventing periodontal disease (Szwetkowski and Falkinham, 2020). They also function as probiotics in the intestines and play a crucial role in maintaining human health (Szwetkowski and Falkinham, 2020). *Bdellovibrio* has the ability to inhibit the formation of multidrug-resistant and even pandrug-resistant bacteria, as well as their biofilms (Szwetkowski and Falkinham, 2020). Furthermore, it does not contribute to the development of bacterial drug resistance (Szwetkowski and Falkinham, 2020), making it advantageous as a potential new biocide (Szwetkowski and Falkinham, 2020). However, the practical application of *Bdellovibrio* faces challenges in terms of its survival. The increased abundance of *Bdellovibrio* observed after chlorogenic acid intervention may pave the way for its future widespread utilization, offering new possibilities for its effective use.

In this study, we observed an increase in the abundance of *Bacteroides* after intervention with chlorogenic acid. *Bacteroides* is a group of pleomorphic non-spore forming Gram-negative anaerobic bacteria (Wexler, 2007). *Bacteroides* is a commonly found resident of the human microbiota, typically inhabiting the intestine, mouth, upper respiratory tract, and reproductive tract of humans and animals. It is considered an important genus that forms the foundation of the gut ecosystem (Zafar and Saier, 2021). They have a symbiotic relationship with humans. *Bacteroides* are usually symbionts that are believed to contribute to the hosts nutritional status as well as mucosal and systemic immunity (Neish, 2009). At the same time, *Bacteroides* are significant clinical pathogens and are found in most anaerobic infections. However, infections caused by *Bacteroides* generally occur only under specific conditions. Among the various infections caused by *Bacteroides*, *Bacteroides fragilis* is the most common. *Bacteroides fragilis* is commonly found in the human oral cavity, gastrointestinal tract, and female reproductive tract. It is an opportunistic pathogen primarily causing endogenous infections, and can lead to infections in the female reproductive system, digestive system, and central nervous system (Wick and Sears, 2010).

Bacteria belonging to the genus *Streptococcus* are the initial inhabitants of the oral cavity, and can be acquired shortly after birth. As such, they play a vital role in the establishment of the oral microbiota (Abranches et al., 2018). In this study, we observed an increase in the abundance of *Streptococcus* after intervention with chlorogenic acid. After the advent of DNA sequencing, the *Streptococci* was separated into 8 distinct groups: *S.mitis*, *S. sanguinis*, *S. anginosus*, *S. salivarius*, *S. downei*, *S. mutans*, *S. pyogenic*, and *S. bovis* (Richards et al., 2014). Presently, oral *Streptococci* are found in all groups except the pyogenic and bovis groups. However, the distribution of oral streptococcal species differs within various ecological niches of the oral cavity (Frandsen et al., 1991). In healthy individuals, the oral biofilm is essentially governed by symbiotic bacteria, which facilitate an intricate dialog involving the host immune system, maintaining an indispensable equilibrium for human health. Dental health is typically associated with a higher proportion of beneficial symbionts, including *Streptococci gordonii*, *Streptococci sanguinis* and *Streptococci parasanguinis*, although some of them can cause opportunistic diseases in different parts of the body (Abranches et al., 2018).

One study documented that 24 children contracted *M. abscessus* infections after treatment at a dental clinic. Further investigations revealed the presence of *M. abscessus* in both source water and DUWLs at the clinic(Szwetkowski and Falkinham, 2020). In 2016, an outbreak of odontogenic mycobacterial infection affected 71 children in California and was attributed to contamination in DUWLs (Szwetkowski and Falkinham, 2020). *Mycobacteria* possess a strong ability to form biofilms and exhibit resistance to high-level disinfectants such as chlorine-based disinfectants, glutaraldehyde, and phthalaldehyde (Esteban and Garcia-Coca, 2017). Consequently, researchers have emphasized the importance of including nontuberculous mycobacteria (NTM) in the monitoring of DUWLs water quality to ensure clinical safety (Szwetkowski and Falkinham, 2020). In our sample, the initial relative abundance of *Mycobacterium* was 4.62% before the intervention. However, following chlorogenic acid intervention, it exhibited a steady decline starting from the first week until the end of the study, with an average relative abundance of 0.58% (*P* < 0.05) (**Supplementary Figure 7**). This result suggests that chlorogenic acid may be a potential inhibitor of *Mycobacterium* and warrants further investigation.

KEGG functional prediction analysis revealed a significant clustering of pre-intervention samples in terms of human disease and immune system disease function, indicating a decrease in the pathogenicity of the bacterial community within DUWLs following chlorogenic acid intervention (**Figure 5**). Furthermore, in the subsequent COG functional prediction analysis, we observed a decrease in the relative abundance of COG1309, COG0596, and COG1960, and an increase in the relative abundance of COG0534 after chlorogenic acid treatment (**Figure 6**). COG1309, identified as a transcriptional regulator of the TetR gene family, is involved in immune response and drug resistance in *Mycobacterium* (Jiang et al., 2015). TetR proteins regulate various physiological and metabolic processes, including antibiotic biosynthesis, drug efflux, morphological differentiation, and pathogenicity (Ramos et al., 2005). COG0596 is a hydrolase or acyltransferase protein belonging to the α/β hydrolase superfamily (Ramos et al., 2005), while the PE-PPE domain is a characteristic feature of this protein family in *Mycobacteria* (Hotelier et al., 2004; Singh et al., 2010; Sultana et al, 2011). COG1960, on the other hand, is a member of the acyl-CoA dehydrogenase family, with similar structural features to FadE5 in *Mycobacterium* (Chen et al., 2020). Studies have demonstrated that these two proteins play crucial roles in mycobacterial lipid metabolism, influencing the virulence and drug resistance of *Mycobacterium* (Chen et al., 2020). These results are in line with the observed decrease in the relative abundance of the aforementioned mycobacterial proteins, indicating that chlorogenic acid may inhibit the proliferation, pathogenicity, and drug resistance of *Mycobacteria* by modulating the transcription of these proteins. Bacterial resistance, especially multidrug resistance, poses a significant challenge in the control of nosocomial infections. COG0534, which was identified in *Vibrio*, functions as a Na-driven multidrug efflux pump (Morita et al., 2000), and cellular drug efflux is one of the key mechanisms contributing to antibacterial resistance (Nikaido, 1996; Mine et al., 1998; Zgurskaya and Nikaido, 1999). In the present study, the relative abundance of COG0534 significantly increased after the intervention with chlorogenic acid, suggesting that the enhanced drug efflux function could be responsible for the observed increase in relative abundance of certain bacteria following treatment with chlorogenic acid.

## Conclusion

This study presents, for the first time, an exploration of the effects of chlorogenic acid treatment on the diversity and abundance of bacterial communities in DUWLs before and after a 3-month period. The results reveal that the core bacterial communities remain intact during chlorogenic acid treatment. A total of 1,877 different OTUs, 316 different genera, and 11 diverse phyla were identified. The dominant phyla in DUWLs were *Proteobacteria*, *Firmicutes*, *Bacteroidetes*, and *Actinobacteria*. *Methylobacterium* and *Bdellovibrio* played a crucial role in distinguishing between the groups. We also observed that chlorogenic acid has a dual impact on the bacterial community in DUWLs. Specifically, chlorogenic acid showed significant inhibitory properties against opportunistic pathogens such as *Mycobacterium*, while also promoting the growth of diverse probiotic strains including *Alloprevotella*, *Roseburia*, and *Blautia*. In conclusion, this study contributes to our understanding of the pathogenesis of oral clinical practice-related infections and provides a new perspective for the prevention and control of oral outpatient infections.

## Data availability statement

The datasets presented in this study can be found in online repositories. The names of the repository/repositories and accession number(s) can be found below: NCBI database, the accession number is BioProject ID: PRJNA958473.

## Author contributions

NL: Conceptualization, Validation, Writing-original draft. Q-M C: Data curation. N-Y H: Formal analysis. S-lJ, F-QC, Q-QH, FY: Methodology. C-Z H: Conceptualization, Visualization, Supervision, Polishing the article.

## Supplementary material

Supplementary Figure 1 Graph of alpha violinplot representing the bacterial community diversity and richness in each DUWLs. PD whole-tree A, Shannon B, chao1 C, observed species D.

Supplementary Figure 2 Barplot of top thirty phyla under chlorogenic acid intervention.

Supplementary Figure 3 Random forest analysis to identify 30 genera species importance point map.

Supplementary Figure 4 KEGG_L3 difference results cluster heatmap.

Supplementary Figure 5 Relative abundance of Methylobacterium under chlorogenic acid intervention.

Supplementary Figure 6 Relative abundance of Bdellovibrio under chlorogenic acid intervention.

Supplementary Figure 7 Relative abundance of Mycobacterium under chlorogenic acid intervention.

Supplementary Table 1 Bacterial contamination status before and after intervention.

Supplementary Table 2 Comparison of Colony number (CFU/ML) among groups. A: The difference between chlorogenic acid and 3% hydrogen peroxide is statistically significant; B: The difference between chlorogenic acid and distilled water is statistically significant; C: The difference between 3% hydrogen peroxide and distilled water is statistically significant.
