# Supplementary figures and images for: Pyrosequencing analysis of bacterial community changes in dental unit waterlines after chlorogenic acid treatment
Source: Front Cell Infect Microbiol. 2024 Jan 17;14:1303099. doi: 10.3389/fcimb.2024.1303099 (PMC10828043; doi:10.3389/fcimb.2024.1303099)

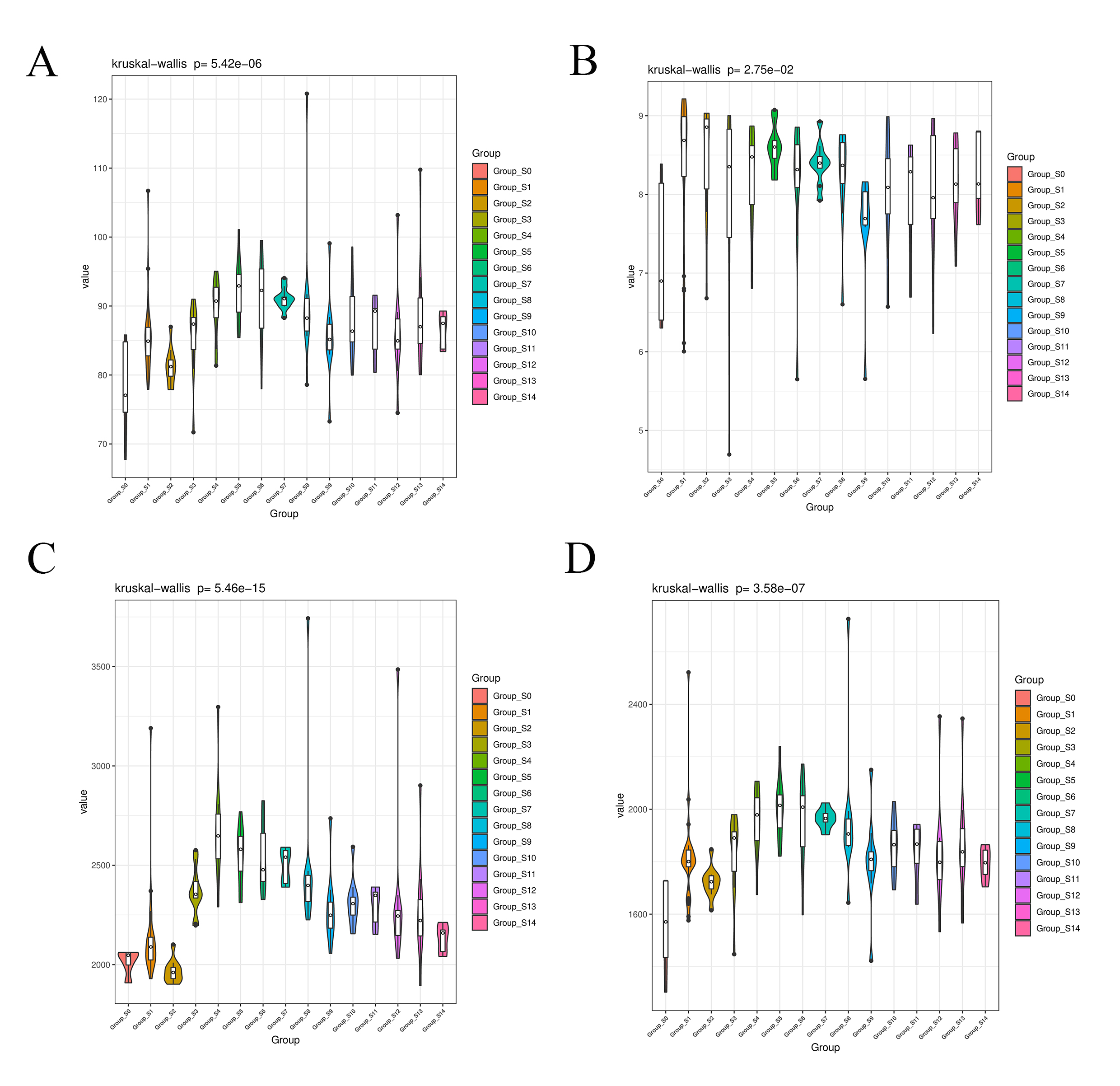

Supplement: Supplementary Figure 1 — Graph of alpha violinplot representing the bacterial community diversity and richness in each DUWLs. PD whole-tree A, Shannon B, chao1 C, observed species D. [file Image_1.jpeg]

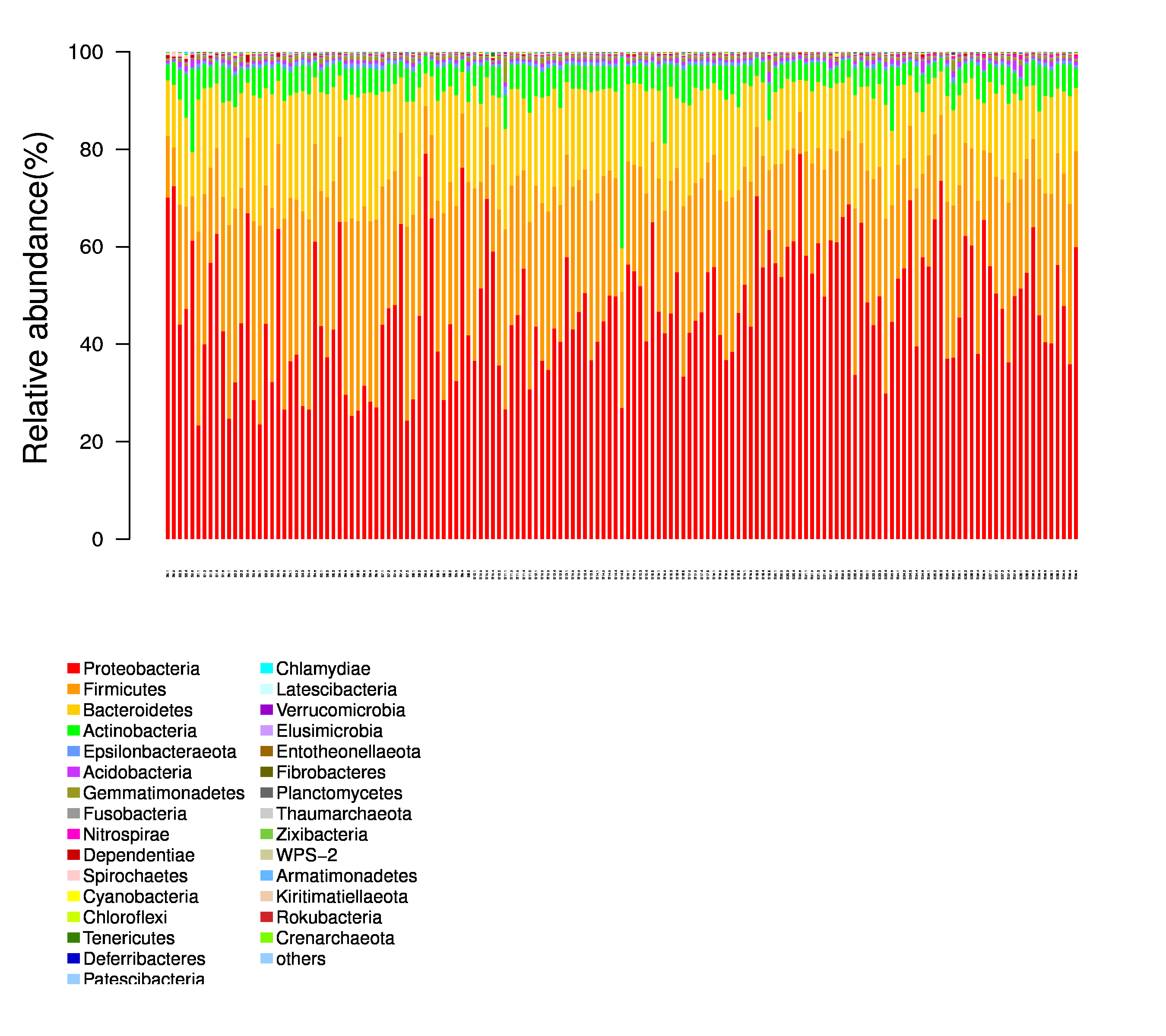

Supplement: Supplementary Figure 2 — Barplot of top thirty phyla under chlorogenic acid intervention. [file Image_2.jpeg]

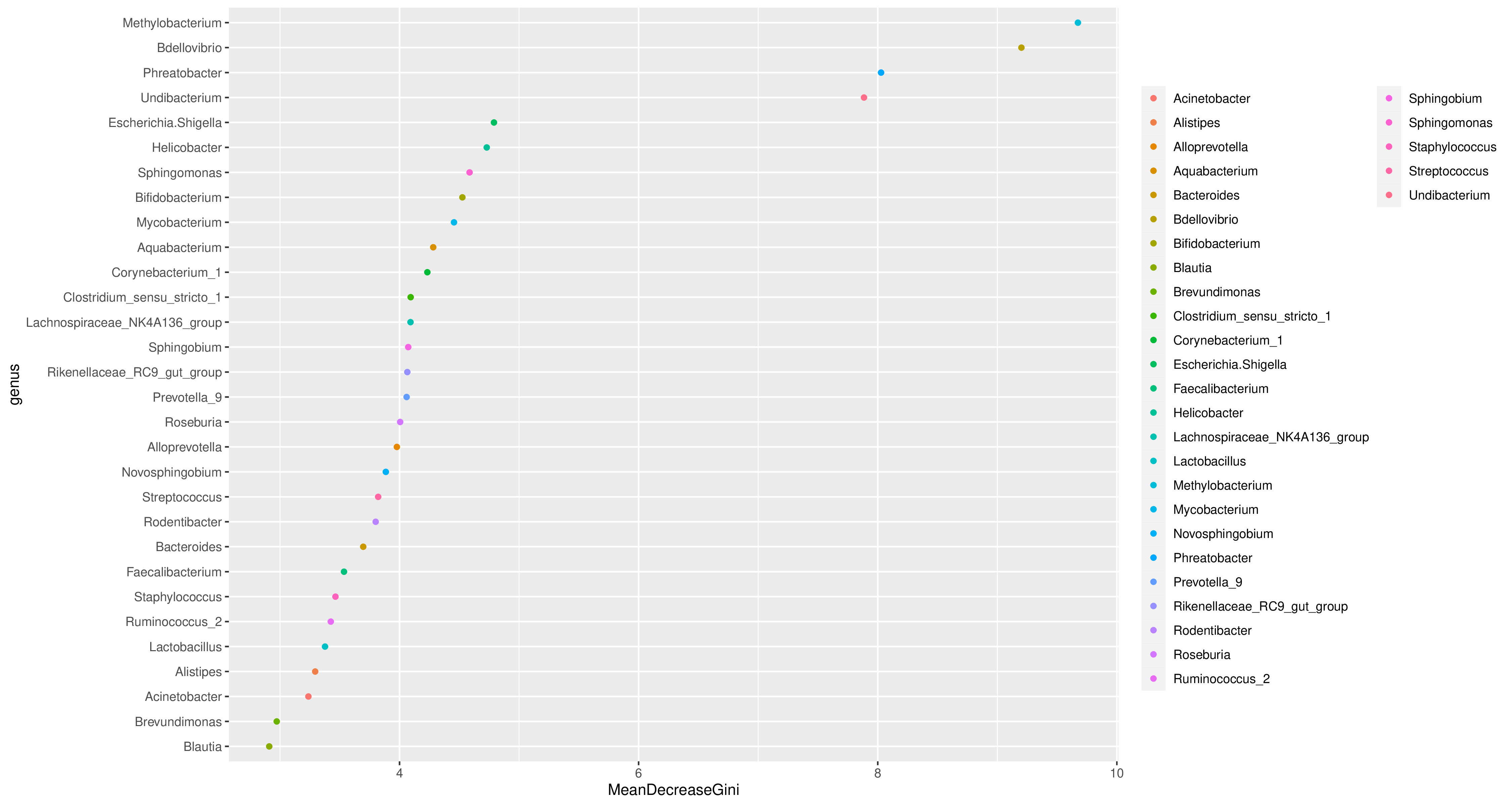

Supplement: Supplementary Figure 3 — Random forest analysis to identify 30 genera species importance point map. [file Image_3.jpeg]

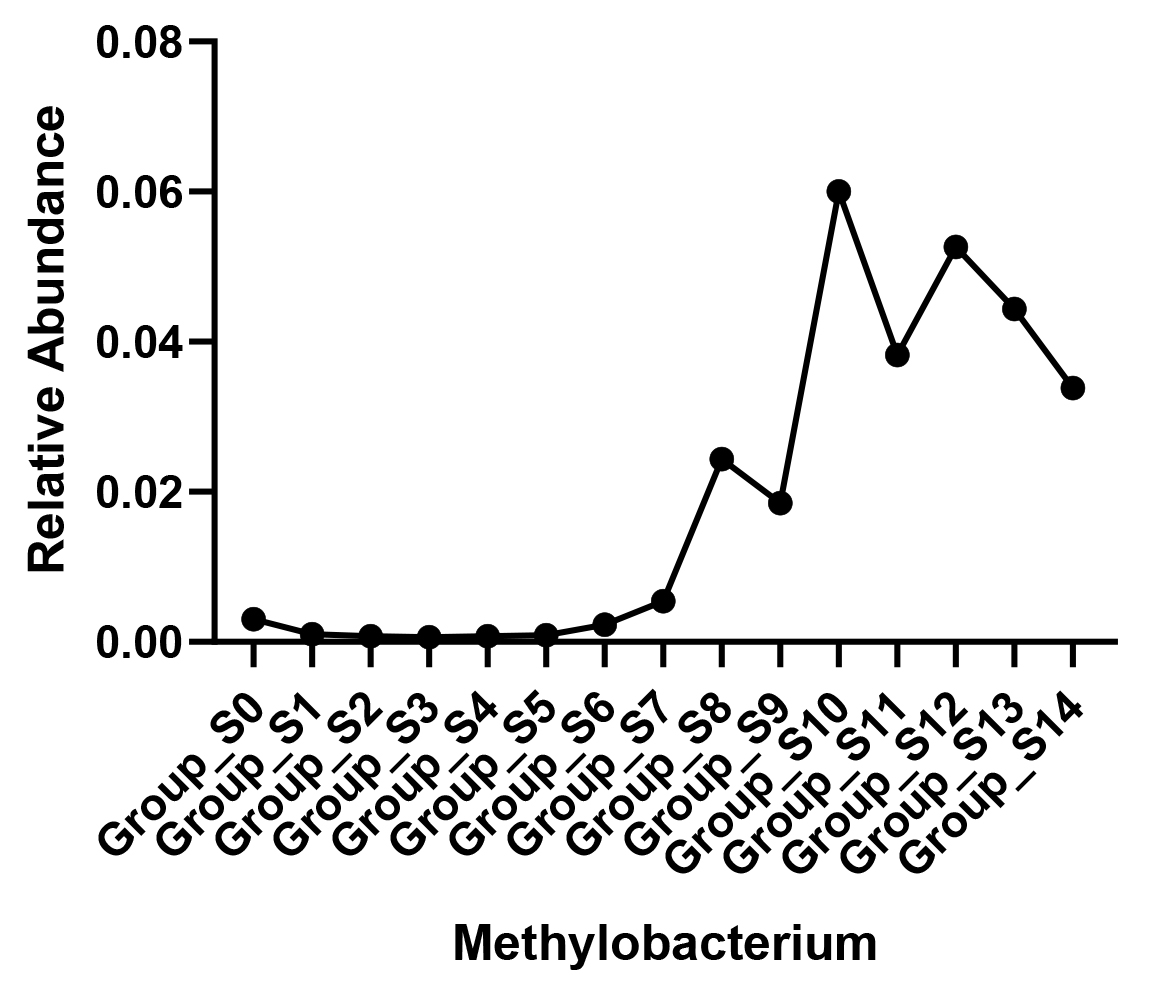

Supplement: Supplementary Figure 5 — Relative abundance of Methylobacterium under chlorogenic acid intervention. [file Image_5.jpeg]

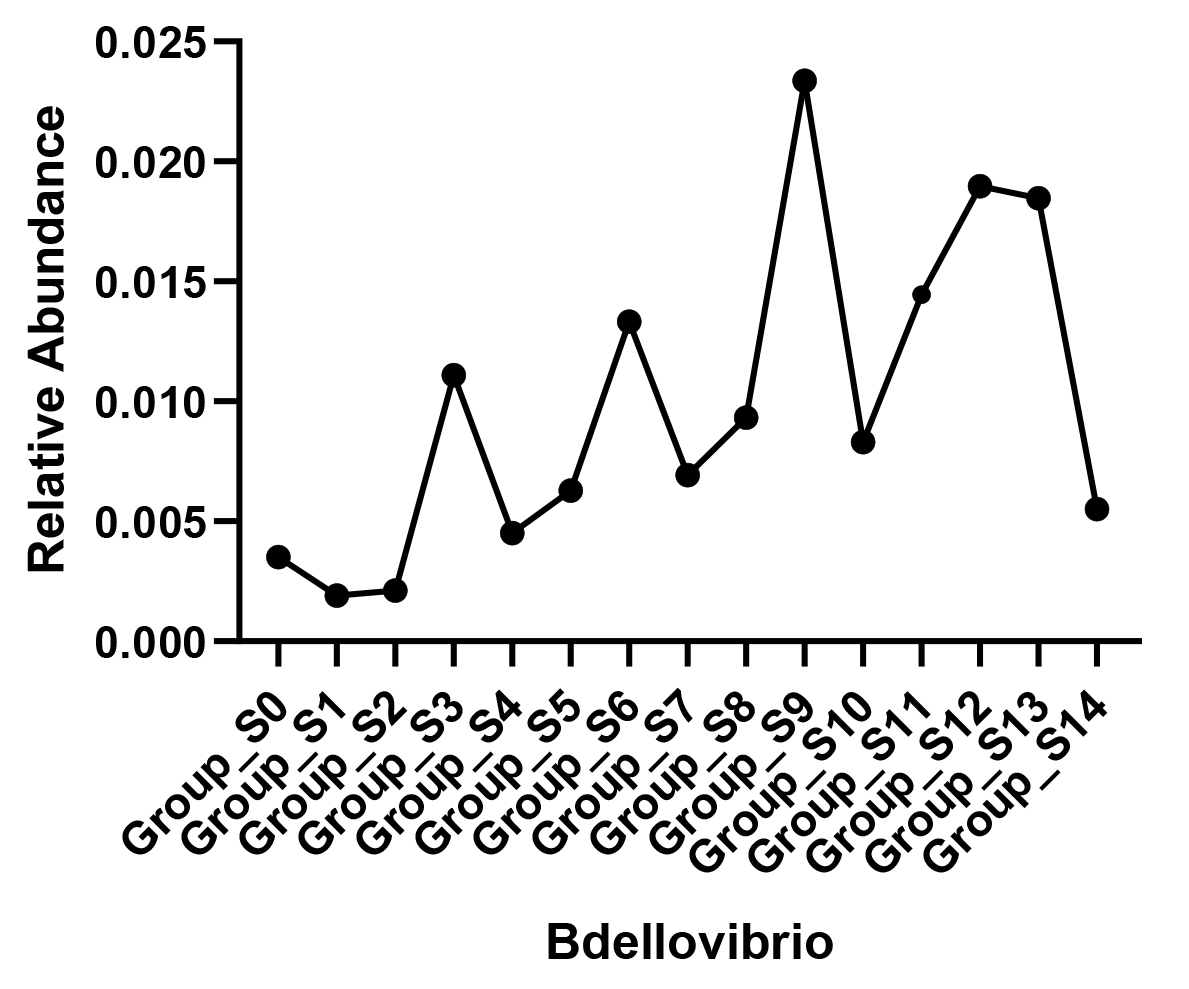

Supplement: Supplementary Figure 6 — Relative abundance of Bdellovibrio under chlorogenic acid intervention. [file Image_6.jpeg]

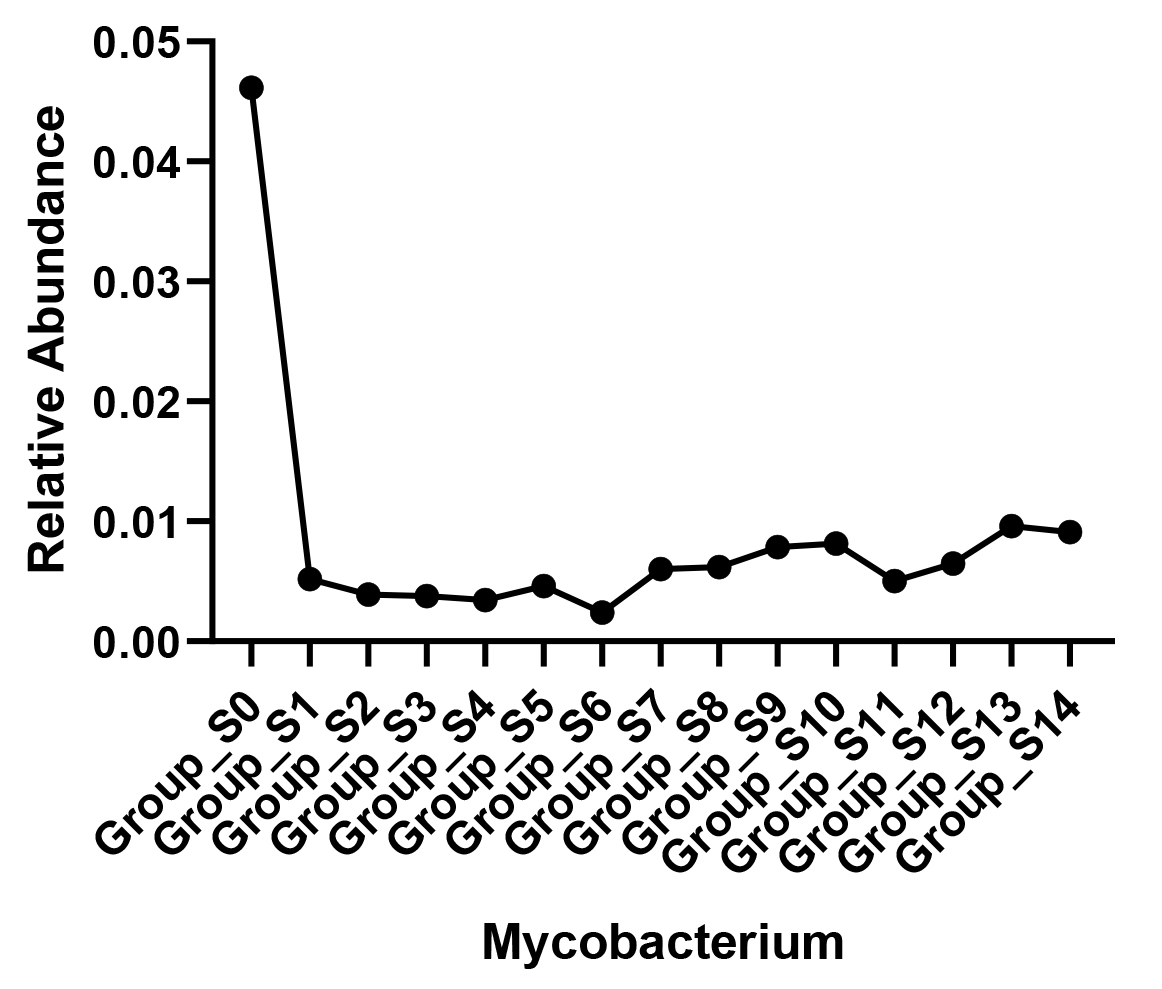

Supplement: Supplementary Figure 7 — Relative abundance of Mycobacterium under chlorogenic acid intervention. [file Image_7.jpeg]
